# Supplementary material for: Therapeutic Effect of a Newly Isolated Lytic Bacteriophage against Multi-Drug-Resistant Cutibacterium acnes Infection in Mice
Source: Int J Mol Sci. 2021 Jun 29;22(13):7031. doi: 10.3390/ijms22137031 (PMC8268795; doi:10.3390/ijms22137031)
Supplement: Supplementary file 1 [file ijms-22-07031-s001.zip › ijms-1245106-supplementary.pdf]

Supplementary Materials

Article

Therapeutic effect of a newly isolated lytic bacteriophage against multi-drug resistant Cutibacterium acnes infection in mice

Ho Yin Pekkle Lam [1,2], Meng-Jiun Lai [3], Ting-Yu Chen [3], Wen-Jui Wu [3], Shih-Yi Peng [1,2,*], Kai-Chih Chang [3,4,*]

[1] Institute of Medical Sciences, Tzu Chi University, Hualien 970, Taiwan; pekklelavabo@gmail.com (H.Y.P.L.)

[2] Department of Biochemistry, School of Medicine, Tzu Chi University, Hualien 970, Taiwan

[3] Department of Laboratory Medicine and Biotechnology, Tzu Chi University, Hualien 970, Taiwan; monjou@mail.tcu.edu.tw (M.-J.L.); 104323103@gms.tcu.edu.tw (T.-Y.C.); w200811@mail.tcu.edu.tw (W.-J.W)

[4] Department of Laboratory Medicine, Buddhist Tzu Chi General Hospital, Hualien 970, Taiwan

* Correspondence: pengsy@mail.tcu.edu.tw (S.-Y.P.); kaichih@mail.tcu.edu.tw (K.-C.C.)

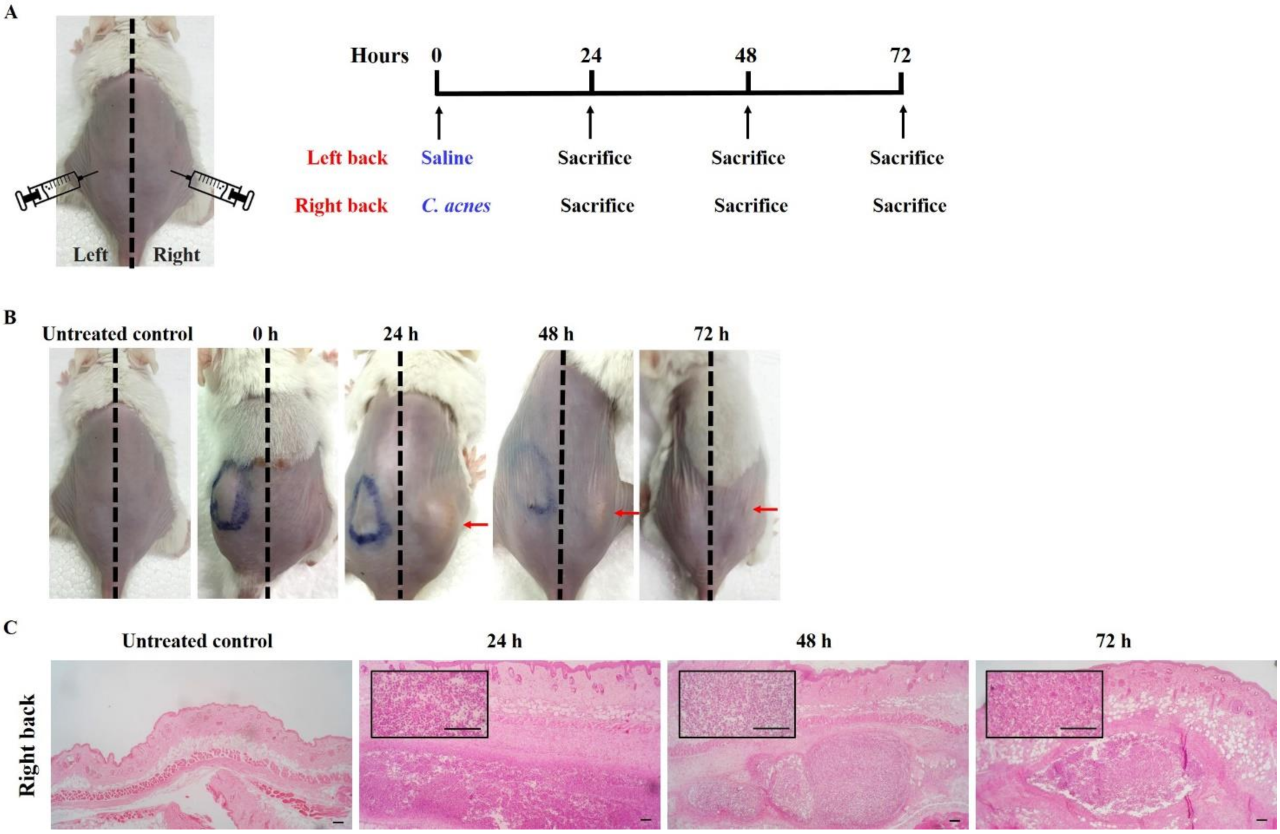

Figure S1: Mice injected with saline and *C. acnes*. (A) Experimental design scheme. (B) Phenotype of mice injected with saline or *C. acnes*. (C) Representative H&E-stained images showing the histological change of the treated mice. Images are shown at 40× and 100× magnification with scale bar = 100 μm.

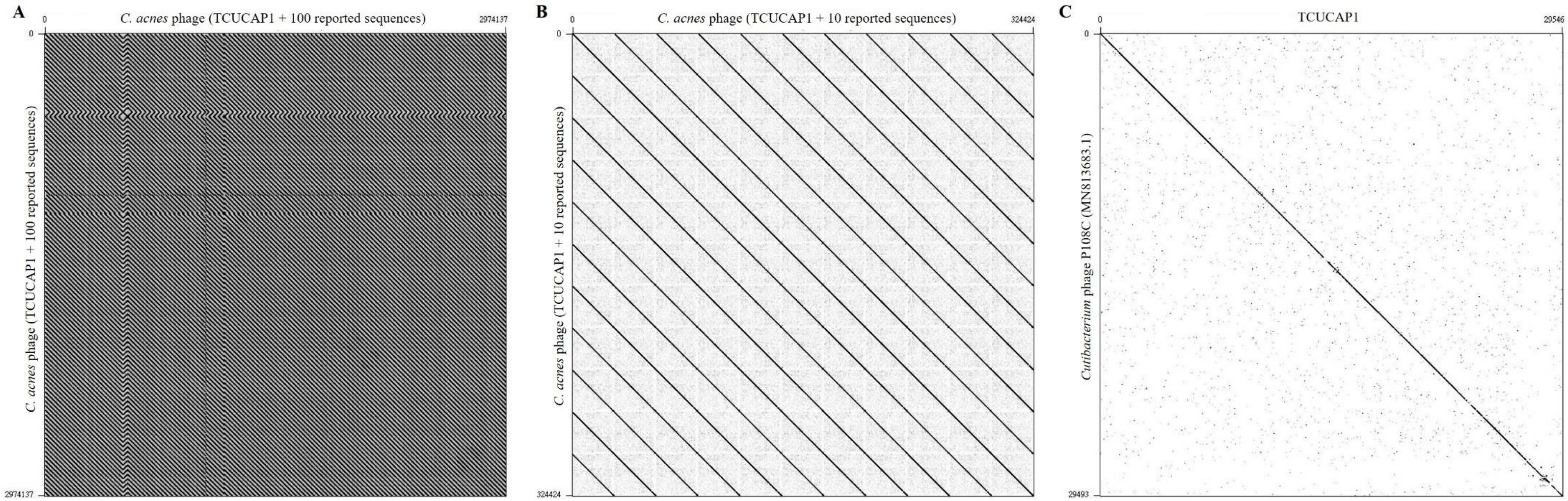

Figure S2: Dot plot nucleotide sequence comparisons of TCUCAP1 and other *C. acnes* phages. Each was compared with itself using the dot plot program Gepard.

Table S1. Functional classification of 56 ORFs in the TCUCAP1 genome

Label	Start	Stop	Length (nt/aa)	Product	Function protein	Accession	Query Cover	E value	Identidy
P2_1	2	88	87/29	Hypothetical protein	-	-	-	-	-
P2_2	60	386	327/109	Putative excinuclease	*Propionibacterium* phage PHL112N00	YP_008531545.1	99.00%	2.00E-68	97.22%
P2_3	391	1902	1512/504	Terminase large subunit	*Cutibacterium* phage P108C	QHB37093.1	99.00%	0	99.01%
P2_4	1899	3224	1326/442	Portal protein	*Cutibacterium* phage P109C	QHB37094.1	99.00%	0	100.00%
P2_5	3231	3986	756/252	Hypothetical protein	*Propionibacterium* phage pa63	AUV62248.1	99.00%	0	99.20%
P2_6	4097	4654	558/186	Scaffold protein	*Propionibacterium* phage PHL009M11	YP_009147229.1	99.00%	2.00E-126	100.00%
P2_7	4661	5608	948/316	Major capsid protein	*Cutibacterium* phage P108C	QHB37097.1	99.00%	0	99.37%
P2_8	5652	6113	462/154	Head-to-tail adaptor	*Cutibacterium* phage P108C	QHB37098.1	99.00%	5E-109	100.00%
P2_9	6079	6621	543/181	Hypothetical protein	*Propionibacterium* phage Pacnes P2	ASJ79976.1	27.00%	6.00E-17	82.00%
P2_10	6876	7127	252/84	Minor tail protein	*Propionibacterium* phage Pirate	YP_009159973.1	98.00%	2.00E-50	98.00%
P2_11	7180	7812	633/211	Hypothetical protein	*Cutibacterium acnes*	WP_002518829.1	99.00%	5.00E-150	99.52%
P2_12	7839	8135	297/99	Hypothetical protein ACQ78_gp12	*Propionibacterium* phage PHL070N00	YP_009146905.1	98.00%	8.00E-62	97.96%
P2_13	8234	8521	288/96	Hypothetical protein ACQ85_gp13	*Propionibacterium* phage PHL009M11	YP_009147237.1	98.00%	9.00E-63	98.95%
P2_14	8529	11294	2766/922	Tape measure protein	*Propionibacterium* phage PHL095N00	YP_009153263.1	99.00%	0	97.18%
P2_15	11310	12251	942/314	Minor tail protein	*Cutibacterium* phage P108C	QHB37106.1	99.00%	0	96.81%
P2_16	12259	13416	1158/386	Putative protease	*Propionibacterium* phage PHL152M00	YP_009152422.1	95.00%	0.00E+00	97.01%
P2_17	13433	14251	819/273	Hypothetical protein P751_gp17	*Propionibacterium* phage PHL071N05	YP_008531697.1	99.00%	0	98.16%
P2_18	14293	14556	264/88	Hypothetical protein D291_gp18	*Propionibacterium* phage P100D	YP_006907147.1	98.00%	5.00E-53	96.55%
P2_19	14559	15365	807/269	Minor tail protein	*Propionibacterium* phage Ouroboros	YP_009160297.1	99.00%	2.00E-157	92.91%
P2_20	15586	16266	681/227	Putative amidase	*Propionibacterium* phage PHL115M02	AGI12651.1	99.00%	2.00E-162	97.35%
P2_21	16279	16674	396/132	Holin	*Cutibacterium* phage P108C	QHB37112.1	99.00%	4.00E-86	99.24%
P2_22	16671	16778	108/36	Hypothetical protein	-	-	-	-	-
P2_23	16960	17250	291/97	Gp23	*Propionibacterium* phage PAS50	YP_004414727.1	79.00%	3.00E-27	87.01%
P2_24	17263	17466	204/68	Hypothetical protein	*Propionibacterium* phage PacnesP1	ASJ79917.1	57.00%	3.00E-08	76.92%
P2_25	17632	17922	291/97	Hypothetical protein	*Propionibacterium* phage PacnesP1	ASJ79916.1	73.00%	2.00E-08	69.01%
P2_26	17888	17983	96/32	Hypothetical protein	-	-	-	-	-
P2_27	18517	18717	201/67	Hypothetical protein	*Cutibacterium* phage PAVL34	QPB11818.1	65.00%	1.00E-09	68.18%
P2_28	18976	19143	168/56	Hypothetical protein	*Cutibacterium* phage PAVL33	QPB11771.1	98.00%	3.00E-10	61.82%
P2_29	19128	19292	165/55	Hypothetical protein	-	-	-	-	-
P2_30	19700	19864	165/55	Hypothetical protein	-	-	-	-	-
P2_31	19885	20202	318/106	Hypothetical protein	-	-	-	-	-
P2_32	20145	20285	141/47	Hypothetical protein	*Cutibacterium* phage PAVL33	QPB11775.1	91.00%	5.00E-08	65.12%
P2_33	20526	20765	240/80	Hypothetical protein	-	-	-	-	-
P2_34	20722	20928	207/69	Hypothetical protein	-	-	-	-	-
P2_35	21032	21184	153/51	Hypothetical protein	-	-	-	-	-
P2_36	21488	21592	105/35	Hypothetical protein	*Cutibacterium* phage FD3	QPB11625.1	97.00%	4.00E-10	91.18%

P2_37	21959	22093	135/45	Hypothetical protein	*Cutibacterium* phage PAVL34	QPB11828.1	95.00%	1.00E-14	83.72%
P2_38	22142	22390	249/83	Hypothetical protein	*Cutibacterium* phage PAVL21	QPB11724.1	44.00%	2.00E-12	86.49%
P2_39	22494	22586	93/31	Hypothetical protein	*Cutibacterium* phage PAVL21	QPB11725.1	96.00%	7.00E-11	96.67%
P2_40	22605	22706	102/34	Hypothetical protein	*Cutibacterium* phage PAVL21	QPB11726.1	97.00%	3.00E-11	84.85%
P2_41	22748	22855	108/36	Hypothetical protein	*Cutibacterium* phage FD1	QPB11524.1	97.00%	5.00E-13	94.29%
P2_42	22881	23285	405/135	Hypothetical protein	*Cutibacterium* phage PAVL34	QPB11832.1	21.00%	0.002	82.76%
P2_43	23944	24117	174/58	Hypothetical protein	-	-	-	-	-
P2_44	24636	24809	174/58	Hypothetical protein	*Cutibacterium* phage PAVL21	QPB11732.1	94.00%	3.00E-21	77.19%
P2_45	24951	25118	168/56	Hypothetical protein	-	-	-	-	-
P2_46	25248	25397	150/50	Hypothetical protein	-	-	-	-	-
P2_47	25593	25934	342/114	Hypothetical protein	*Cutibacterium* phage PAVL34	QPB11838.1	32.00%	4.00E-08	75.68%
P2_48	25942	26124	183/61	Hypothetical protein	-	-	-	-	-
P2_49	26286	26408	123/41	Hypothetical protein	*Cutibacterium* phage FD1	QPB11532.1	90.00%	2.00E-10	83.78%
P2_50	26412	26720	309/103	Hypothetical protein	*Cutibacterium* phage PAVL21	QPB11738.1	53.00%	6.00E-19	83.64%
P2_51	26906	27043	138/46	Hypothetical protein	*Propionibacterium* phage pa29399-1-D_1	AUV62200.1	86.00%	3.00E-13	82.50%
P2_52	27342	27500	159/53	Hypothetical protein P759_gp43	*Propionibacterium* phage PHL010M04	YP_008531678.1	60.00%	4.00E-09	87.50%
P2_53	28899	29054	156/52	Hypothetical protein	*Cutibacterium* phage FD2	QPB11588.1	48.00%	1.00E-05	84.00%
P2_54	29176	29310	135/45	Hypothetical protein	*Propionibacterium* phage PacnesP1	ASJ79897.1	97.00%	1.00E-14	79.55%
P2_55	29294	29467	174/58	Hypothetical protein	*Cutibacterium* phage FD2	QPB11590.1	77.00%	3.00E-20	91.11%
P2_56	29406	29546	141/47	Hypothetical protein	-	-	-	-	-
